# Supplementary material for: Cancer-Selective Treatment of Cancerous and Non-Cancerous Human Cervical Cell Models by a Non-Thermally Operated Electrosurgical Argon Plasma Device
Source: Cancers (Basel). 2020 Apr 23;12(4):1037. doi: 10.3390/cancers12041037 (PMC7226384; doi:10.3390/cancers12041037)
Supplement: Supplementary file 1 [file cancers-12-01037-s001.pdf]

cancers

*Supplementary Materials*

# Cancer-Selective Treatment of Cancerous and Non-Cancerous Human Cervical Cell Models by a Non-Thermally Operated Electrosurgical Argon Plasma Device

**Lukas Feil, André Koch, Raphael Utz, Michael Ackermann, Jakob Barz, Matthias Stope, Bernhard Krämer, Diethelm Wallwiener, Sara Y. Brucker and Martin Weiss**

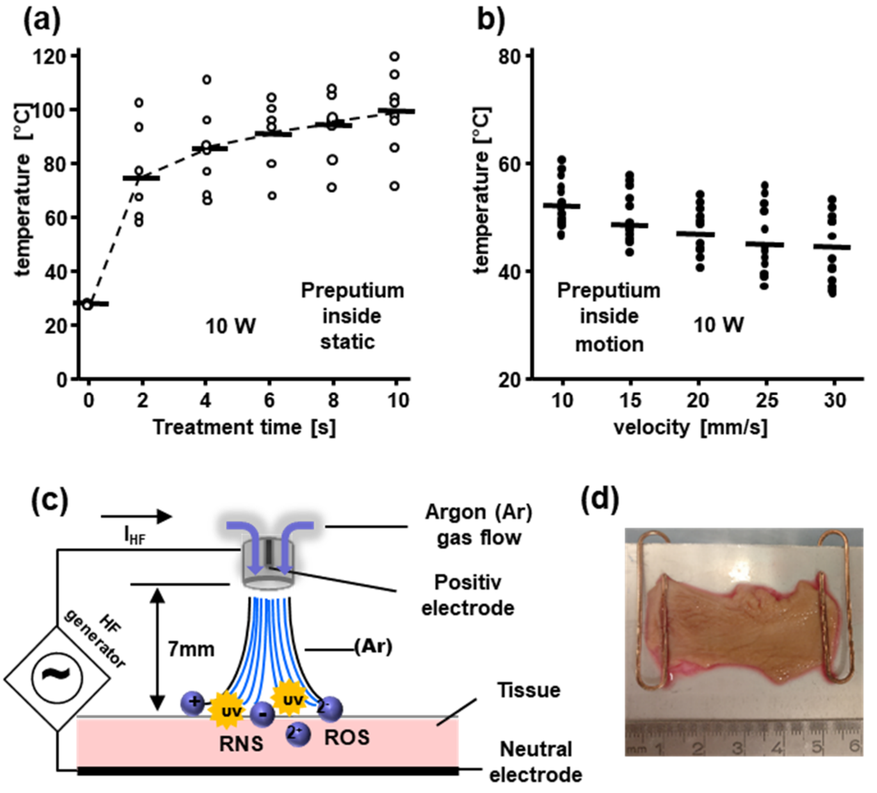


**Figure S1.** Infrared thermography and OES measurement of the non-thermally operated MABS. Fresh preputial tissue samples (non-keratinized squamous epithelium) (d) were analyzed during static (a) and velocity-dependent (b) treatment with MABS. Results are expressed as the mean ± SD. Schematic reconstruction of the experimental setup (c).

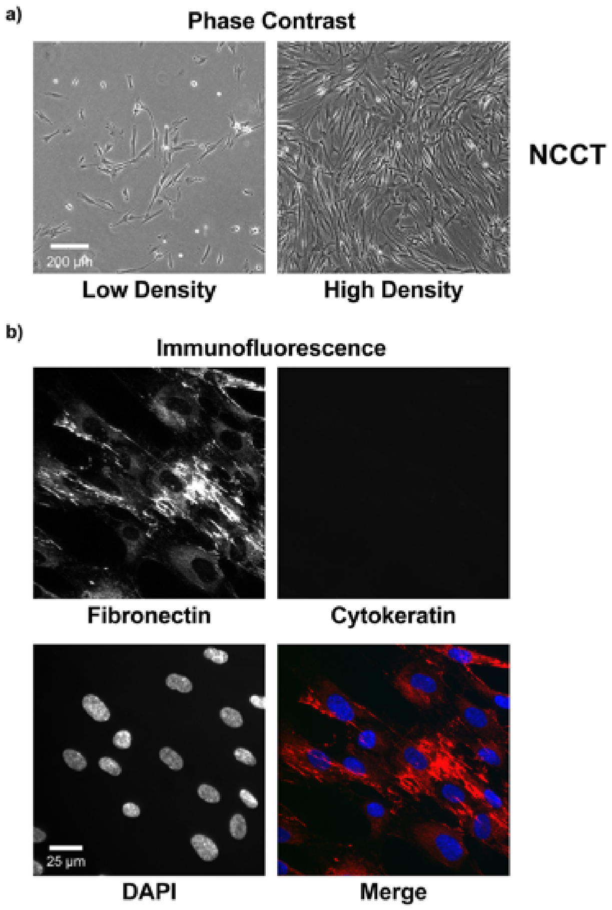


**Figure S2.** Characterization of NCCT cells. a) Phase contrast images of a 2D-culture of low (left) and high density (right). The scale bar indicates 200 µm. b) Immunofluorescent images of NCCT cells stained with Fibronectin (red), Cytokeratin (green), and DAPI (blue) and a merge of all three colors showing the mesenchymal nature of the cells. The scale bar indicates 25 µm.

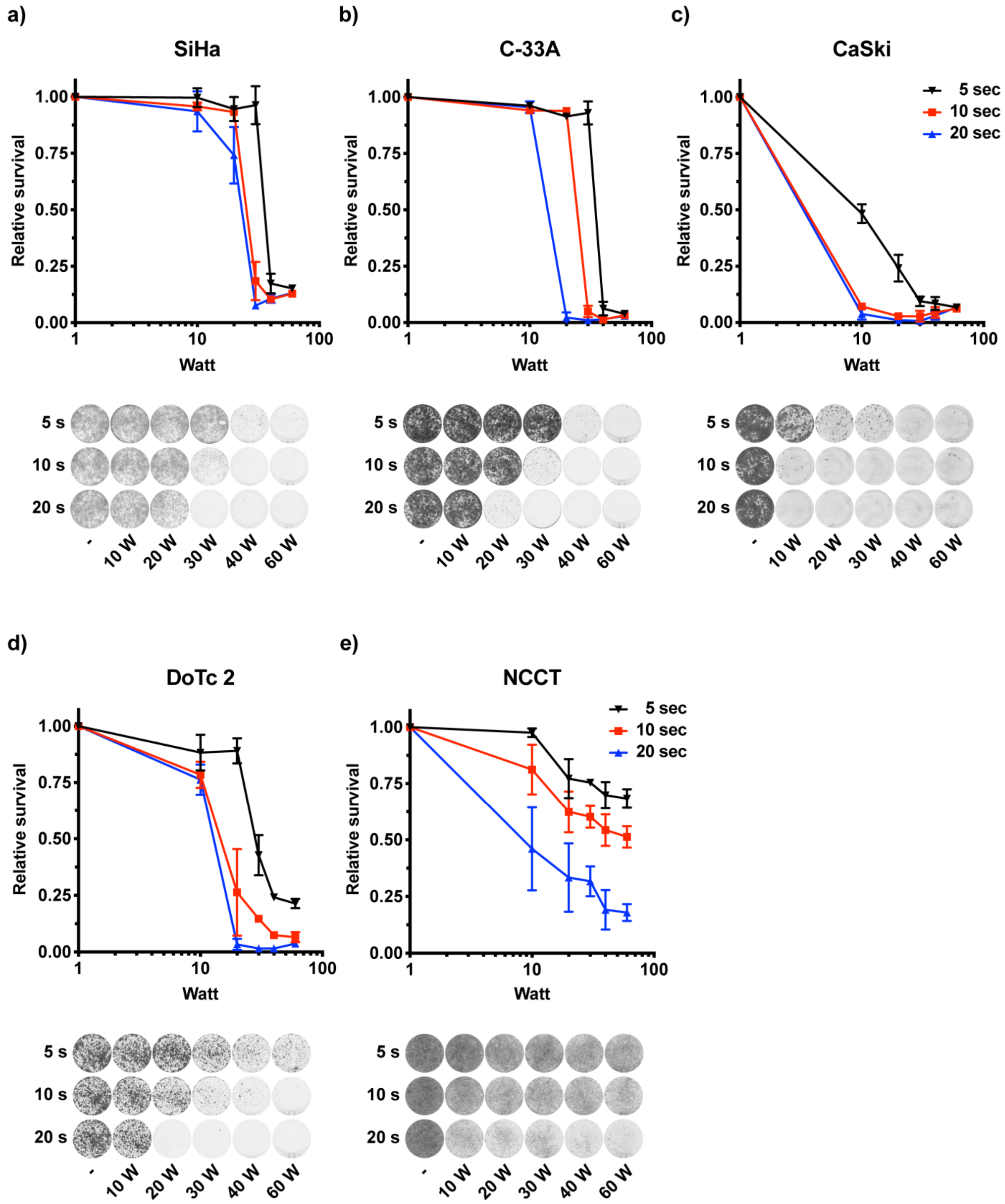


**Figure S3.** NCCT cells are less sensitive to MABS compared to CC cells. Relative survival plots of the CC cell lines. SiHa (a), C-33 A (b), Ca Ski (c), DoTc 2 (d) or NCCT cells fixed 6 days after MABS treatment with increasing watt power for 5 s (black), 10 s (red), and 20 s (blue). Shown is the average of three independent experiments, including standard deviations. Below the survival plots are the crystal violet stainings of one representative proliferation assay shown.

© 2020 by the authors. Licensee MDPI, Basel, Switzerland. This article is an open access article distributed under the terms and conditions of the Creative Commons Attribution (CC BY) license (http://creativecommons.org/licenses/by/4.0/).
